# Tetra­ammine­(carbonato-κ^2^ *O*,*O*′)cobalt(III) nitrate: a powder X-ray diffraction study

**DOI:** 10.1107/S1600536813017522

**Published:** 2013-06-29

**Authors:** Armel Le Bail

**Affiliations:** a Université du Maine, Institut des Molécules et des Matériaux du Mans, CNRS UMR 6283, 72085 Le Mans, France

## Abstract

Practical chemistry courses at universities very frequently propose the synthesis and characterization of [Co(CO_3_)(NH_3_)_4_]NO_3_, but this goal is never achieved since students only obtain the hemihydrated form. The anhydrous form can be prepared, however, and its structure is presented here. Similar to the hemihydrate form, the anhydrous phase contains the Co^III^ ion in an octahedral O_2_N_4_ coordination by a chelating carbonate group and four ammine ligands. The structure reveals an intricate array of N—H⋯O hydrogen bonds involving both the chelating and the non-chelating O atoms of the carbonate ligand as hydrogen-bond acceptors of the amine H atoms, which are also involved in hydrogen-bonding inter­actions with the nitrate O atoms. The structure of the anhydrous form is close to that of the hemihydrate phase, suggesting a probable topotactic reaction with relatively small rotations and translations of the [Co(CO_3_)(NH_3_)_4_]^+^ and NO_3_^−^ groups during the dehydration process, which produces an unusual volume increase of 4.3%.

## Related literature

For the crystal structure of the hemihydrate, see: Bernal & Cetrullo (1990 ▶); Junk & Steed (1999 ▶); Christensen & Hazell (1999 ▶). For the synthesis of the hemihydrate, see: Schlessinger (1960 ▶). For powder diffraction indexing figures of merit, see: de Wolff (1968 ▶); Smith & Snyder (1979 ▶). For profile refinement by the Le Bail method, see: Le Bail (2005 ▶). For preferred orientation correction, see: Dollase (1986 ▶).

## Experimental

### 

#### Crystal data

[Co(CO_3_)(NH_3_)_4_]NO_3_
*M* *_r_* = 249.09
Monoclinic, 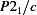

*a* = 7.8520 (6) Å
*b* = 6.7922 (5) Å
*c* = 17.5394 (9) Å
β = 95.440 (3)°
*V* = 931.21 (11) Å^3^
*Z* = 4
Cu *K*α radiation, λ = 1.5418 Å
*T* = 293 K
flat sheet, 8 × 8 mm

#### Data collection

Siemens D500 diffractometer
Specimen mounting: packed on the holder
Data collection mode: reflection
Scan method: step
2θ_min_ = 6.001°, 2θ_max_ = 79.961°, 2θ_step_ = 0.020°

#### Refinement

*R*_p_ = 0.054
*R*_wp_ = 0.072
*R*_exp_ = 0.025
*R*_Bragg_ = 0.030
*R*(*F*) = 0.026
χ^2^ = 8.123
3751 data points
114 parameters
55 restraints
H atoms treated by a mixture of independent and constrained refinement

### 

Data collection: *DIFFRAC-AT* (Siemens & Socabim, 1993 ▶); cell refinement: *McMaille* (Le Bail, 2004 ▶); data reduction: *DIFFRAC-AT*; program(s) used to solve structure: *ESPOIR* (Le Bail, 2001 ▶); program(s) used to refine structure: *FULLPROF* (Rodriguez-Carvajal, 1993 ▶); molecular graphics: *DIAMOND* (Brandenburg, 1999 ▶); software used to prepare material for publication: *PLATON* (Spek, 2009 ▶).

## Supplementary Material

Crystal structure: contains datablock(s) global, I. DOI: 10.1107/S1600536813017522/vn2075sup1.cif

Rietveld powder data: contains datablock(s) I. DOI: 10.1107/S1600536813017522/vn2075Isup2.rtv

Additional supplementary materials:  crystallographic information; 3D view; checkCIF report

## Figures and Tables

**Table 1:** Selected bond lengths (Å)

Co1—O1	1.928 (8)
Co1—O2	1.913 (9)
Co1—N1	2.004 (8)
Co1—N2	1.953 (9)
Co1—N3	1.953 (9)
Co1—N4	2.012 (8)

**Table 2:** Hydrogen-bond geometry (Å, °)

*D*—H⋯*A*	*D*—H	H⋯*A*	*D*⋯*A*	*D*—H⋯*A*
N1—H1⋯O4^i^	0.952 (10)	2.476 (15)	3.392 (16)	161.3 (11)
N1—H2⋯O4^ii^	0.949 (14)	2.481 (16)	3.218 (13)	134.5 (11)
N2—H4⋯O3^iii^	0.962 (13)	2.400 (15)	3.261 (13)	148.8 (12)
N2—H5⋯O3^iv^	0.962 (11)	2.190 (15)	3.021 (12)	144.0 (12)
N2—H6⋯O4^ii^	0.962 (10)	2.511 (16)	3.331 (13)	143.1 (11)
N3—H7⋯O3^iv^	0.957 (13)	2.448 (15)	3.278 (13)	145.0 (11)
N3—H8⋯O6^v^	0.963 (13)	2.151 (17)	2.762 (16)	120.0 (12)
N3—H8⋯O2^vi^	0.963 (13)	2.496 (17)	3.184 (12)	128.3 (10)
N3—H9⋯O5	0.969 (10)	2.102 (14)	3.047 (14)	164.4 (13)
N4—H10⋯O3^iv^	0.946 (10)	2.180 (11)	3.069 (12)	156.1 (12)
N4—H11⋯O1^iii^	0.948 (13)	1.993 (12)	2.911 (10)	162.5 (12)
N4—H12⋯O2^vi^	0.955 (13)	2.231 (11)	3.059 (10)	144.5 (11)

Symmetry codes: (i) 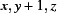; (ii) 
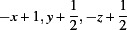
; (iii) 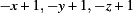; (iv) 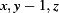; (v) 
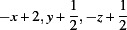
; (vi) 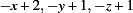.

**Fig. 1.**
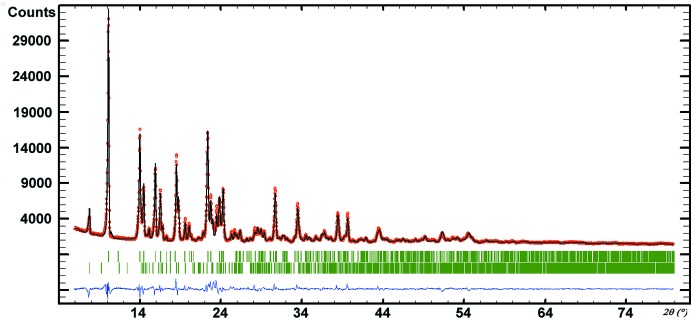
Final Rietveld plot. Observed data points are indicated by dots, the best-fit profile (upper trace) and the difference pattern (lower trace) are solid lines. The two series of vertical bars indicate the position of Bragg peaks for the anhydrous (upper line) and hemihydrate (lower line) forms.

**Fig. 2.**
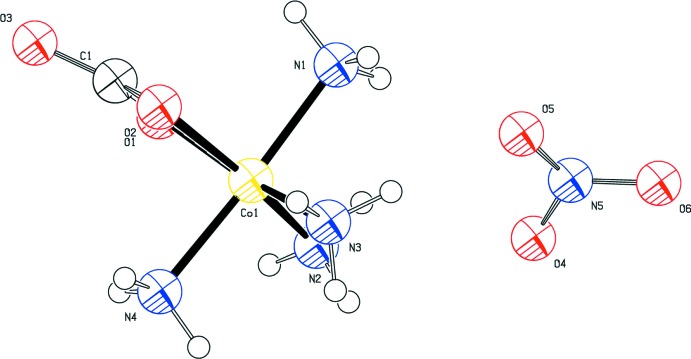
ORTEP plot of the [Co(NH3)4CO3]+ and [NO3]- groups showing the atom numbering. Displacement spheres are drawn at the 50% probability level and H atoms are shown as small spheres of arbitrary radii.

**Fig. 3.**
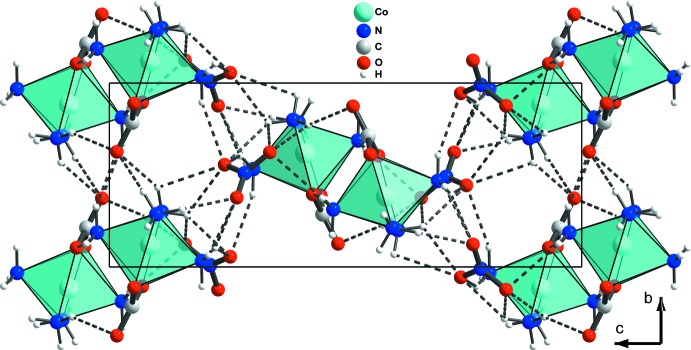
Projection of the structure of the anhydrous phase along the a axis with representation of the intricate N—H···O hydrogen bonding scheme.

**Fig. 4.**
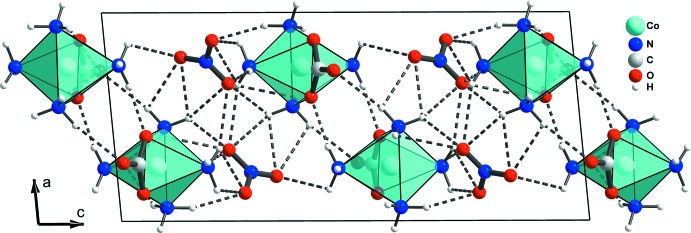
Projection of the structure of the anhydrous phase along the b axis, showing the alternate stacking of layers of [NO3]- and [Co(NH3)4CO3]+ groups along c.

**Fig. 5.**
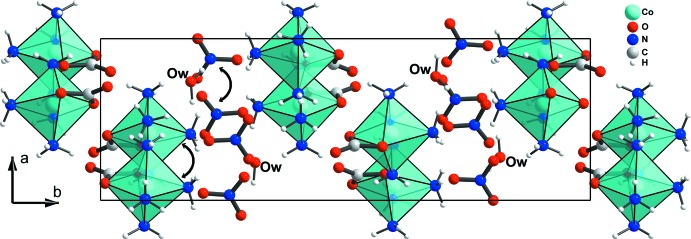
Projection of the structure of the hemihydrate phase along the c axis. In the topotactic dehydration hypothesis, this c axis has to be divided by two (Z = 8 → 4). The two curved arrows relate two tetraamine-carbonato-cobalt and two nitrate groups which are separated by ~1/2c and should translate in inverse direction along a in order to attain their superposition in projection onto the ab plane and also should finally rotate, making both Figures 5 and 4 identical at the end of the dehydration process. Water molecules are indicated by 'Ow'.

## supplementary crystallographic information

### Comment

The synthesis and characterization of [Co(NH_3_)_4_CO_3_]NO_3_ is a frequent choice for practical chemistry courses at universities. Under the conditions described, students only obtain the hemihydrated form whose crystal structure was determined three times from single-crystal data (Bernal & Cetrullo, 1990; Junk & Steed, 1999; Christensen & Hazell, 1999). In the latter study, a thermogravimetric analysis has shown that the anhydrous form is produced at 125°C before the complete decomposition into Co_3_O_4_ at 230°C. Reproducing this TGA experiment complemented by a DSC measurement, it was decided to isolate the anhydrous form and to solve its structure by powder diffraction methodologies since no suitable single-crystal could be obtained.

It was impossible to obtain a sample free of the hemihydrate phase in air, even when stopping the DSC scan at 180°C. The anhydrous form is hygroscopic, but the rehydration stops before being complete. Therefore, rather than realising a thermodiffraction analysis under controlled atmosphere, a powder diffractogram was recorded on a stabilized mixture of the anhydrous and hemihydrate forms. Indexing was realized using the *McMaille* software (Le Bail, 2004), applied to 20 peak positions at 2Θ < 34°, leading to a monoclinic cell with figures of merit M20 = 29 (de Wolff, 1968), F20 = 51 (0.006,65) (Smith & Snyder, 1979). Intensities were extracted by the Le Bail (2005) method using the *Fullprof* software (Rodriguez-Carvajal, 1993), refining only a scale factor for the impurity phase (the hemihydrate) having its atomic coordinates fixed to those of the most accurate single-crystal study (Bernal and Cetrullo, 1990). The structure solution was undertaken in direct space, using the *ESPOIR* software (Le Bail, 2001), moving independently the [NO_3_]^-^ and the [Co(NH_3_)_4_CO_3_]^+^ groups (12 degrees of freedom) by a Monte Carlo process up to find a satisfying *R* value. The final Rietveld refinement was done using the *Fullprof* software (Fig. 1); the hemihydrate fraction in the biphased powder was estimated to be 19%. A displacement sphere plot is shown in Fig. 2.

Each molecular group was refined with a large number of restraints in order to keep its geometry acceptable, *i.e.* similar as that in the hemihydrate phase; this makes any discussion about the internal geometry senseless. Discussing the intermolecular distances is possible for the *N*(amine)···O contacts, but not for the H···O ones since the H atom positions are inherited from the tetraamine-carbonato-cobalt group of the hemihydrate used at the structure solution stage. However, even if certainly inaccurate, the hydrogen bonding scheme looks acceptable, allthough a few N—H···O angles are below 120° but at the same time belonging to the shortest N···O distances (which are in the 2.76–3.39 Å range, compared to 2.89–3.09 Å in the hemihydrate). Anyway, there is no doubt that the hydrogen bonding scheme in the anhydrous form, similar to that found in the hemihydrate phase, is responsable for the three-dimensional cohesion of the crystal structure by a complex array of bonds from the amine H atoms to both the chelating and nonchelating O atoms of the carbonato ligands and to the nitrate O atoms (Figures 3 and 4).

The close similarity between the crystal structures of the anhydrous form and the hemihydrate phase suggests a topotactic dehydration by relatively small moves of the tetraammine-carbonato-cobalt and nitrate groups. As such the long axis is retained (*b*_hemi_ = 22.673 Å in the hemihydrate and *c*_anh_ = 17.539 Å in the anhydrous form) and the *a* axis as well (*a*_hemi_ = 7.496 Å and *a*_anh_ = 7.852 Å), whereas the *Z* variation from 8 to 4 is the result of halving the *c*_hemi_ parameter (*c*_hemi_ = 10.513 Å and *b*_anh_ = 6.7922 Å). The [Co(NH_3_)_4_CO_3_]^+^ and [NO_3_]^-^ groups stay approximately at the same place since a similar stacking in alternate layers of these groups is observed along *b*_hemi_ and *c*_anh_. Moreover, the tetraamine-carbonato-cobalt groups correspond almost two by two in the hemihydrate phase by a translation of 1/2c (Fig. 5 to be compared to Fig. 4), and the same is true for the nitrate groups. The two curved arrows in Fig. 5 show that each group in these pairs of groups at \sim 1/2*c* apart may displace half their distance in opposite direction along the *a* axis in order to attain a quasi perfect superposition in projection onto the *ab* plane allowing then to divide *c* by two when the water has gone away. Additional rotations of the groups and cell parameters adjustments are also necessary to reproduce the final arrangement in the anhydrous form (Fig. 4).

Unusual is the fact that the dehydration produces a 4.3% volume increase which may be due to a relaxation after the disappearance of the strong hydrogen bonds previously associated to the water molecules. This would perhaps explain the uncomplete rehydration in air because the possibility of water penetration would be stopped soon at the crystallite surface once rehydrated, due to the volume retraction. Voids were found in neither the anhydrous nor hemihydrate phase (*PLATON* ; Spek, 2009).

### Experimental

The hemihydrate was prepared according to the method published by Schlessinger (1960), and then dehydrated.

### Refinement

The Rietveld refinement was performed using intramolecular interatomic distance restraints (±0.01 Å) as obtained from the hemihydrate single-crystal study (Bernal and Cetrullo, 1990): N—H = 0.96 Å, H—H = 1.58 Å, Co—N = 1.955 Å, Co—O = 1.91 Å, Co—H = 2.44 Å, C—O = 1.308 Å, C=O = 1.23 Å, N—O = 1.229 Å and O—O = 2.13 Å in the nitrate group and 2.25 Å or 2.152 Å in the carbonate group. Such conditons impose almost a rigid body refinement. A strong preferred orientation along the *c* axis for the anhydrous form and the *b* axis for the hemihydrate was detected and treated by the March-Dollase (Dollase, 1986) approach. Isotropic atomic displacements were restrained to be the same during refinements inside of the three groups NO_3_, CO_3_, CoN_4_, and were fixed for the hydrogen atoms.

### Crystal data

**Table:**

[Co(CO_3_)(NH_3_)_4_]NO_3_	*Z* = 4
*M**_r_* = 249.09	*F*(000) = 512.0
Monoclinic, *P*2_1_/*c*	*D*_x_ = 1.777 Mg m^−^^3^
Hall symbol: -P 2ybc	Cu *K*α radiation, λ = 1.5418 Å
*a* = 7.8520 (6) Å	*T* = 293 K
*b* = 6.7922 (5) Å	Particle morphology: Fine powder
*c* = 17.5394 (9) Å	purple
β = 95.440 (3)°	flat sheet, 8 × 8 mm
*V* = 931.21 (11) Å^3^	

### Data collection

**Table:**

Siemens D500 diffractometer	Data collection mode: reflection
Radiation source: X-ray tube	Scan method: step
Graphite monochromator	2θ_min_ = 6.001°, 2θ_max_ = 79.961°, 2θ_step_ = 0.020°
Specimen mounting: packed on the holder	

### Refinement

**Table:**

*R*_p_ = 5.371	Profile function: pseudoVoigt
*R*_wp_ = 7.237	114 parameters
*R*_exp_ = 2.536	55 restraints
*R*_Bragg_ = 3.00	H atoms treated by a mixture of independent and constrained refinement
*R*(*F*) = 2.64	(Δ/σ)_max_ = 0.03
χ^2^ = 8.123	Background function: interpolated
3751 data points	Preferred orientation correction: March-Dollase correction along direction [001], refined parameter : 0.634(2)

### Special details

**Table:**

Geometry. Bond distances, angles *etc*. have been calculated using the rounded fractional coordinates. All su's are estimated from the variances of the (full) variance-covariance matrix. The cell e.s.d.'s are taken into account in the estimation of distances, angles and torsion angles

### Fractional atomic coordinates and isotropic or equivalent isotropic displacement parameters (Å^2^)

**Table:**

	*x*	*y*	*z*	*U*_iso_*/*U*_eq_	
Co1	0.7395 (5)	0.3827 (5)	0.41424 (15)	0.061 (2)*	
O1	0.5942 (8)	0.6047 (12)	0.4304 (6)	0.026 (3)*	
O2	0.8681 (8)	0.6112 (12)	0.4476 (6)	0.026 (3)*	
O3	0.7215 (14)	0.8735 (12)	0.4842 (6)	0.026 (3)*	
N1	0.7399 (10)	0.4675 (12)	0.3048 (4)	0.061 (2)*	
N2	0.5497 (10)	0.2010 (12)	0.3890 (4)	0.061 (2)*	
N3	0.9350 (11)	0.2129 (12)	0.4005 (4)	0.061 (2)*	
N4	0.7470 (10)	0.3120 (12)	0.5259 (4)	0.061 (2)*	
C1	0.7279 (11)	0.7129 (15)	0.4519 (12)	0.026 (3)*	
O4	0.6593 (14)	−0.056 (2)	0.2560 (5)	0.065 (4)*	
O5	0.8659 (19)	0.123 (2)	0.2302 (7)	0.065 (4)*	
O6	0.7854 (19)	−0.1237 (16)	0.1602 (5)	0.065 (4)*	
N5	0.766 (2)	−0.010 (3)	0.2132 (8)	0.065 (4)*	
H1	0.7181 (18)	0.6054 (9)	0.3038 (7)	0.0633*	
H2	0.6522 (14)	0.3947 (18)	0.2765 (6)	0.0633*	
H3	0.8506 (10)	0.437 (2)	0.2899 (7)	0.0633*	
H4	0.4590 (12)	0.2308 (19)	0.4204 (7)	0.0633*	
H5	0.5904 (15)	0.0688 (10)	0.3983 (8)	0.0633*	
H6	0.5091 (16)	0.2178 (19)	0.3359 (4)	0.0633*	
H7	0.9232 (16)	0.0926 (13)	0.4279 (7)	0.0633*	
H8	1.0386 (10)	0.2804 (16)	0.4190 (8)	0.0633*	
H9	0.9359 (16)	0.1860 (19)	0.3463 (4)	0.0633*	
H10	0.7522 (18)	0.1729 (9)	0.5281 (7)	0.0633*	
H11	0.6458 (12)	0.3646 (19)	0.5434 (6)	0.0633*	
H12	0.8476 (12)	0.3735 (19)	0.5498 (6)	0.0633*	

### Geometric parameters (Å, º)

**Table:**

Co1—O1	1.928 (8)	N1—H2	0.949 (14)
Co1—O2	1.913 (9)	N1—H1	0.952 (10)
Co1—N1	2.004 (8)	N1—H3	0.954 (12)
Co1—N2	1.953 (9)	N2—H4	0.962 (13)
Co1—N3	1.953 (9)	N2—H5	0.962 (11)
Co1—N4	2.012 (8)	N2—H6	0.962 (10)
O1—C1	1.307 (13)	N3—H9	0.969 (10)
O2—C1	1.308 (12)	N3—H7	0.957 (13)
O3—C1	1.232 (16)	N3—H8	0.963 (13)
O4—N5	1.218 (19)	N4—H10	0.946 (10)
O5—N5	1.22 (2)	N4—H11	0.948 (13)
O6—N5	1.23 (2)	N4—H12	0.955 (13)
			
O1—Co1—O2	67.9 (3)	Co1—N1—H1	106.5 (9)
O1—Co1—N1	88.3 (4)	Co1—N2—H4	109.1 (9)
O1—Co1—N2	94.5 (4)	H4—N2—H5	110.6 (13)
O1—Co1—N3	164.4 (4)	H4—N2—H6	109.5 (13)
O1—Co1—N4	90.4 (4)	H5—N2—H6	110.4 (13)
O1—Co1—C1	33.9 (3)	Co1—N2—H5	108.5 (9)
O2—Co1—N1	90.6 (4)	Co1—N2—H6	108.7 (10)
O2—Co1—N2	162.1 (4)	Co1—N3—H9	108.1 (10)
O2—Co1—N3	96.8 (4)	Co1—N3—H7	109.1 (10)
O2—Co1—N4	86.0 (4)	Co1—N3—H8	109.0 (9)
O2—Co1—C1	33.9 (3)	H7—N3—H9	110.0 (13)
N1—Co1—N2	92.0 (3)	H8—N3—H9	109.6 (13)
N1—Co1—N3	88.8 (3)	H7—N3—H8	110.9 (13)
N1—Co1—N4	176.6 (4)	Co1—N4—H10	106.0 (9)
N1—Co1—C1	89.9 (6)	Co1—N4—H12	105.6 (9)
N2—Co1—N3	101.0 (4)	H10—N4—H11	113.4 (14)
N2—Co1—N4	91.2 (3)	Co1—N4—H11	106.1 (9)
N2—Co1—C1	128.3 (4)	H11—N4—H12	112.1 (12)
N3—Co1—N4	91.6 (3)	H10—N4—H12	112.9 (14)
N3—Co1—C1	130.7 (4)	O5—N5—O6	122.0 (16)
N4—Co1—C1	87.3 (6)	O4—N5—O5	120.4 (15)
Co1—O1—C1	90.7 (6)	O4—N5—O6	116.6 (17)
Co1—O2—C1	91.3 (6)	Co1—C1—O3	169.0 (15)
Co1—N1—H2	106.5 (9)	O1—C1—O2	110.1 (10)
Co1—N1—H3	106.5 (9)	O1—C1—O3	124.3 (10)
H1—N1—H2	112.5 (13)	O2—C1—O3	124.4 (11)
H1—N1—H3	112.1 (14)	Co1—C1—O1	55.4 (5)
H2—N1—H3	112.2 (13)	Co1—C1—O2	54.7 (5)
			
O2—Co1—O1—C1	−1.0 (10)	N1—Co1—C1—O2	−91.3 (10)
N1—Co1—O1—C1	−92.4 (10)	N2—Co1—C1—O1	−5.4 (13)
N2—Co1—O1—C1	175.8 (10)	N2—Co1—C1—O2	176.3 (7)
N4—Co1—O1—C1	84.5 (10)	N3—Co1—C1—O1	175.5 (7)
O1—Co1—O2—C1	1.0 (10)	N3—Co1—C1—O2	−2.9 (13)
N1—Co1—O2—C1	88.9 (10)	N4—Co1—C1—O1	−94.8 (9)
N3—Co1—O2—C1	177.8 (10)	N4—Co1—C1—O2	86.9 (10)
N4—Co1—O2—C1	−91.1 (10)	Co1—O1—C1—O2	1.4 (14)
O1—Co1—C1—O2	−178.4 (17)	Co1—O1—C1—O3	−166.6 (18)
O2—Co1—C1—O1	178.4 (17)	Co1—O2—C1—O1	−1.4 (14)
N1—Co1—C1—O1	87.1 (9)	Co1—O2—C1—O3	166.6 (17)

### Hydrogen-bond geometry (Å, º)

**Table:**

*D*—H···*A*	*D*—H	H···*A*	*D*···*A*	*D*—H···*A*
N1—H1···O4^i^	0.952 (10)	2.476 (15)	3.392 (16)	161.3 (11)
N1—H2···O4^ii^	0.949 (14)	2.481 (16)	3.218 (13)	134.5 (11)
N1—H3···O5	0.954 (12)	2.384 (19)	2.900 (16)	113.6 (11)
N2—H4···O6^ii^	0.962 (13)	2.479 (17)	2.942 (16)	109.4 (10)
N2—H4···O3^iii^	0.962 (13)	2.400 (15)	3.261 (13)	148.8 (12)
N2—H5···O3^iv^	0.962 (11)	2.190 (15)	3.021 (12)	144.0 (12)
N2—H6···O4^ii^	0.962 (10)	2.511 (16)	3.331 (13)	143.1 (11)
N2—H6···O6^ii^	0.962 (10)	2.558 (19)	2.942 (16)	104.0 (9)
N3—H7···O3^iv^	0.957 (13)	2.448 (15)	3.278 (13)	145.0 (11)
N3—H8···O6^v^	0.963 (13)	2.151 (17)	2.762 (16)	120.0 (12)
N3—H8···O2^vi^	0.963 (13)	2.496 (17)	3.184 (12)	128.3 (10)
N3—H9···O5	0.969 (10)	2.102 (14)	3.047 (14)	164.4 (13)
N4—H10···O3^iv^	0.946 (10)	2.180 (11)	3.069 (12)	156.1 (12)
N4—H11···O1^iii^	0.948 (13)	1.993 (12)	2.911 (10)	162.5 (12)
N4—H12···O2^vi^	0.955 (13)	2.231 (11)	3.059 (10)	144.5 (11)

Symmetry codes: (i) *x*, *y*+1, *z*; (ii) −*x*+1, *y*+1/2, −*z*+1/2; (iii) −*x*+1, −*y*+1, −*z*+1; (iv) *x*, *y*−1, *z*; (v) −*x*+2, *y*+1/2, −*z*+1/2; (vi) −*x*+2, −*y*+1, −*z*+1.

## References

[bb1] Bernal, I. & Cetrullo, J. (1990). *Struct. Chem.* **1**, 227–234.

[bb2] Brandenburg, K. (1999). *DIAMOND* Crystal Impact GbR, Bonn, Germany.

[bb3] Christensen, A. N. & Hazell, R. G. (1999). *Acta Chem. Scand.* **53**, 399–402.

[bb4] Dollase, W. A. (1986). *J. Appl. Cryst.* **19**, 267–272.

[bb5] Junk, P. C. & Steed, J. W. (1999). *Polyhedron*, **18**, 3593–3597.

[bb6] Le Bail, A. (2001). *Mater. Sci. Forum*, **378**, 65–70.

[bb7] Le Bail, A. (2004). *Powder Diffr.* **19**, 249–254.

[bb8] Le Bail, A. (2005). *Powder Diffr.* **20**, 316–326.

[bb9] Rodriguez-Carvajal, J. (1993). *Physica B*, **192**, 55–69.

[bb10] Schlessinger, G. (1960). *Inorg. Synth.* **6**, 173–175.

[bb11] Siemens & Socabim (1993). *DIFFRAC-AT* Siemens Analytical X-ray Instruments, Inc., Madison, Wisconsin, USA, and Socabim SA, Paris, France.

[bb12] Smith, G. S. & Snyder, R. L. (1979). *J. Appl. Cryst.* **12**, 60–65.

[bb13] Spek, A. L. (2009). *Acta Cryst.* D**65**, 148–155. 10.1107/S090744490804362X PMC2631630 19171970

[bb14] Wolff, P. M. de (1968). *J. Appl. Cryst.* **1**, 108–113.

